# Post-insertion technique to introduce targeting moieties in milk exosomes for targeted drug delivery

**DOI:** 10.1186/s40824-023-00456-w

**Published:** 2023-11-29

**Authors:** Hochung Jang, Hyosuk Kim, Eun Hye Kim, Geonhee Han, Yeongji Jang, Yelee Kim, Jong Won Lee, Sang Chul Shin, Eunice EunKyeong Kim, Sun Hwa Kim, Yoosoo Yang

**Affiliations:** 1 Medicinal Materials Research Center, Biomedical Research Institute, Korea Institute of Science and Technology (KIST), Seoul, 02792 Republic of Korea; 2 Division of Bio-Medical Science and Technology, KIST School, University of Science and Technology, Seoul, 02792 Republic of Korea; 3 Department of Life Sciences, Korea University, Seoul, 02841 Republic of Korea; 4 KU-KIST Graduate School of Converging Science and Technology, Korea University, Seoul, 02841 Republic of Korea; 5 Technological Convergence Center, Research Resources Division, Korea Institute of Science and Technology (KIST), Seoul, 02792 Republic of Korea

**Keywords:** Milk-derived exosome, Surface modification, Post-insertion, Targeted delivery, Antitumor effects

## Abstract

**Background:**

Recently, increased attention has been given on exosomes as ideal nanocarriers of drugs owing to their intrinsic properties that facilitate the transport of biomolecular cargos. However, large-scale exosome production remains a major challenge in the clinical application of exosome-based drug delivery systems. Considering its biocompatibility and stability, bovine milk is a suitable natural source for large-scale and stable exosome production. Because the active-targeting ability of drug carriers is essential to maximize therapeutic efficacy and minimize side effects, precise membrane functionalization strategies are required to enable tissue-specific delivery of milk exosomes with difficulty in post-isolation modification.

**Methods:**

In this study, the membrane functionalization of a milk exosome platform modified using a simple post-insertion method was examined comprehensively. Exosomes were engineered from bovine milk (mExo) with surface-tunable modifications for the delivery of tumor-targeting doxorubicin (Dox). The surface modification of mExo was achieved through the hydrophobic insertion of folate (FA)-conjugated lipids.

**Results:**

We have confirmed the stable integration of functionalized PE-lipid chains into the mExo membrane through an optimized post-insertion technique, thereby effectively enhancing the surface functionality of mExo. Indeed, the results revealed that FA-modified mExo (mExo-FA) improved cellular uptake in cancer cells via FA receptor (FR)-mediated endocytosis. The designed mExo-FA selectively delivered Dox to FR-positive tumor cells and triggered notable tumor cell death, as confirmed by in vitro and in vivo analyses.

**Conclusions:**

This simple and easy method for post-isolation modification of the exosomal surface may be used to develop milk-exosome-based drug delivery systems.

**Graphical Abstract:**

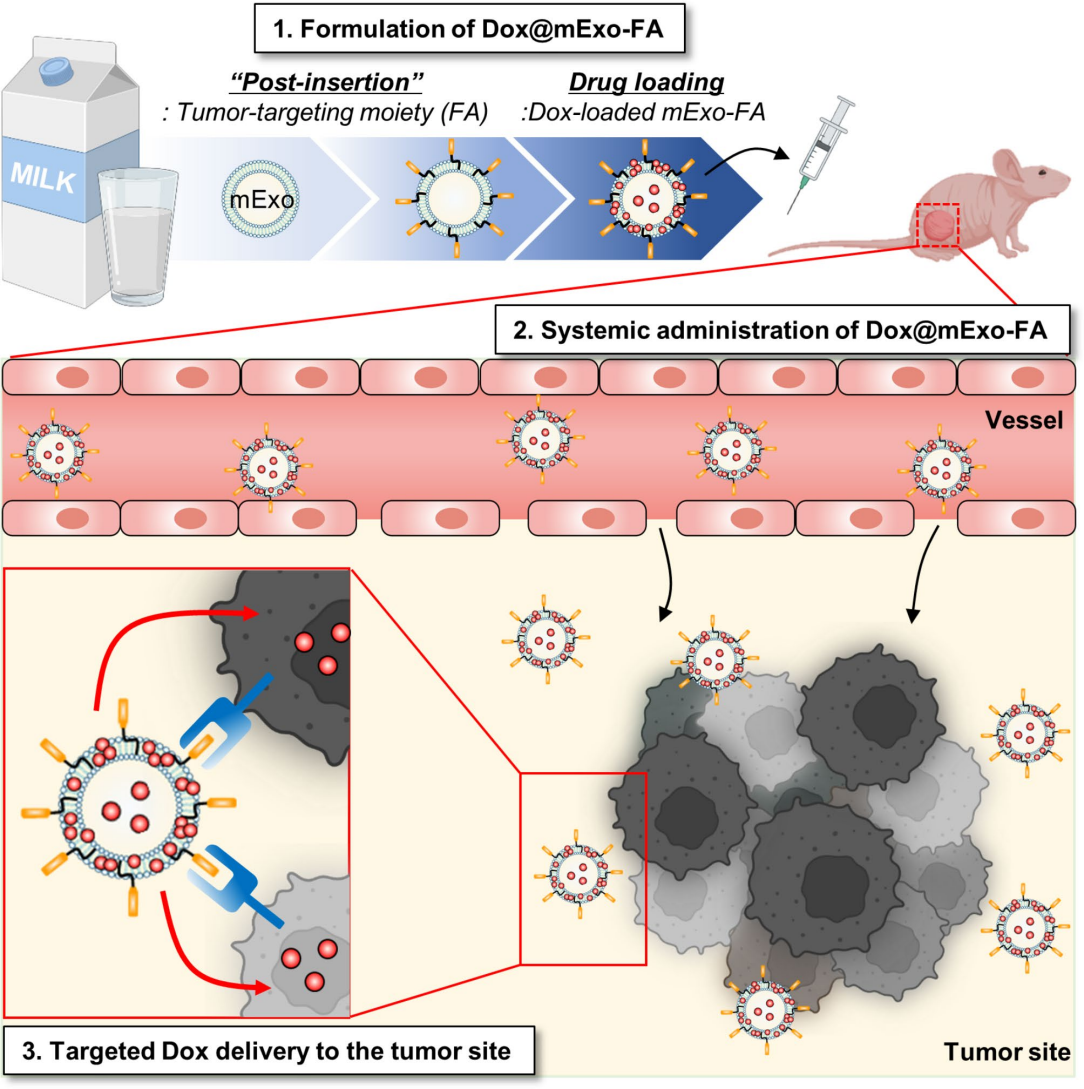

**Supplementary Information:**

The online version contains supplementary material available at 10.1186/s40824-023-00456-w.

## Background

Breastfeeding has nutritional benefits from milk and can boost immunity and hence reduce the risk of developing diseases. In particular, chemoprevention for childhood leukemia and lymphoma [1], as well as the improvement of otitis media [2] and asthma through breastfeeding has been studied extensively [3]. Furthermore, exosomes from bovine milk are significantly enriched in proteins and microRNAs associated with infant growth and immune maturation [4]. Based on these observations, exosomes, important components that exhibit clear functional activity and are present in human and bovine milk, have gained considerable attention. Exosomes are nanosized (30‒150 nm in diameter) particles secreted from all cell types [5]. These naturally occurring nanocarriers that mediate intercellular communication by delivering biomacromolecules can be used for drug delivery [6, 7].

Because the stability of bovine milk-derived exosomes (mExo) has been confirmed, their potential as an alternative drug delivery system has been considered [5, 8–10]. Exosomes derived from cell culture media exhibit favorable advantages for drug delivery, including high biocompatibility, low cytotoxicity, and drug encapsulation ability. However, as achieving high production yields sufficient to conduct preclinical and clinical studies remains challenging [11], exosomes from bovine milk are a promising alternative to cellular exosomes. A previous study demonstrated that approximately 20-fold more exosomes were isolated from milk than from the same volume of cell culture medium [10]. In addition, milk exosomes maintain their physicochemical properties well under the harsh and degrading conditions of the gastrointestinal tract and repeated freeze-thaw cycles [8–10]. These properties of mExo indicate their potential as a drug delivery system in terms of long-term storage, cost-effective production, and safety.

Despite the excellent stability, low immunogenicity, and biocompatibility, additional surface modification is required to employ mExo as drug delivery vehicle [12]. In the present study, a post-insertion method was used for the membrane modification of mExo. Compared with genetic manipulation at the cellular level, membrane post-insertion is a more effective strategy to modify the exosome surface using a simple and flexible approach [13, 14]. Post-insertion is frequently employed to produce ligand-coupled liposomes. In this method, the ligands of interest are first covalently linked to polyethylene glycol‒lipid micelles. Subsequently, the modified micelles are mixed with liposomes, followed by the migration of micelles into the liposomal bilayer. [15]. A functional ligand can be incorporated into the lipid surface via passive, stable hydrophobic insertion of a conjugated lipid tail. In several cases of postmodification using lipid-conjugated functional residues, phospholipids, especially phosphatidylethanolamine (PE), are frequently used as lipid anchors owing to their intrinsic hydrophobic properties that incorporate into the membrane [16–18].

Based on the elastic nature of exosomal membranes, which can be easily incorporated into different lipids, mExo were functionalized for targeted cancer therapy by introducing a lipid-conjugated cancer-targeting moiety, folate (FA), on the mExo surface [19, 20]. To produce surface-modified mExo, a simple post-insertion method was utilized, with some modifications, using functionalized PE lipids. Insertion of exogenous lipids into mExo membranes was validated using three strategies, depending on the functional moiety conjugated to the lipid head group: (i) detection of fluorescence-labeled avidin, which covalently interacted with PE-biotin inserted in the mExo membrane, by native gel electrophoresis; (ii) observation of Flamma 675-azide, which was conjugated by click reaction to Flamma 496-labeled and dibenzocyclooctyne (DBCO)-inserted mExo in cells; and (iii) measurement of FA-specific absorbance intensity.

In the present study, FA-decorated mExo (mExo-FA) were evaluated to confirm whether surface modification can facilitate tumor-targeting doxorubicin (Dox) delivery. The cellular uptake of mExo-FA by cancer cells was enhanced in an FA receptor (FR)-dependent manner. Using Dox-loaded/FA-modified mExo (Dox@mExo-FA), FA-mediated Dox delivery delayed tumor growth in FR-positive HCT116 tumor-bearing mice. Thus, the proposed mExo modification method can provide guidance for incorporating various targeting moieties into milk exosomes, which is necessary for applying milk exosomes as a promising targeted drug delivery platform.

## Methods

### Isolation of exosomes from bovine milk

All batches of mExo were isolated from 400 mL of commercial low-fat milk that was pasteurized at 63 ℃ and free of antibiotics. The entire isolation process was composed of two types of serial centrifugation and conducted at 4 ℃ (Fig. S1). Initially, to separate large-size contaminants [21], including milk fat and cell debris, the milk was centrifuged at 5000 x g for 30 min followed by 12,000 x g for 1 h using an Avanti J-E high-speed centrifuge with a fixed-angle JA-14 rotor (Beckman Coulter). After centrifugation, the supernatant underwent additional filtration using a cell strainer (pore size: 40 μm, SPL) and was stored at ‒20 ℃ until further use. To isolate mExo, centrifuged milk supernatant was subjected to ultracentrifugation at 35,000 x g for 1 h followed by 70,000 x g for 3 h. The supernatant obtained from the previous centrifugation step was then serially filtered by using 0.8, 0.45, and 0.2 μm pore-sized syringe filters (Sartorius) to eliminate the remaining contaminants, including bacteria. The filtered supernatant underwent a final ultracentrifugation step at 100,000 x g for 1 h using an Optima XE-100 with a fixed-angle 45Ti rotor (Beckman Coulter), and the mExo pellet was resuspended in ice-cold 1X phosphate-buffered saline (PBS, Thermo Fisher). To obtain exosome-free milk for validating negative markers of exosome and milk protein contaminants, the supernatant collected from the 100,000 x g centrifugation step was subjected to additional ultracentrifugation at 200,000 x g for 3 h. Before using mExo for each experiment, the total amount of mExo was quantified by using a Pierce BCA protein assay kit (Thermo Fisher) according to the manufacturer’s instructions.

#### Dynamic light scattering analysis

All types of mExo species (mExo, mExo-FA, Dox@mExo, Dox@mExo-FA) were diluted in filtered distilled water (DW; refractive index: 1.330) to a 50 µg/mL (total analysis volume: 1 mL) and loaded into a transparent cuvette. The sample-loaded cuvettes were placed in a dynamic light scattering (DLS) instrument (Zetasizer Nano ZS; Malvern), and the intensity (%) was measured through a five-repeated analysis. All data were collected using Zetasizer software. According to the ISO22412 standard, the z-average and polydispersity index (PDI) values indicate the diameter and dispersity of the mExo species, respectively.

### Transmission electron microscopy

In the experiment, 10 µg of mExo, mExo-FA, and Dox@mExo-FA were suspended in ultrapure-filtered water. Samples (5 µL) were seeded on the carbon film on 200 mesh copper grids (Electron Microscopy Sciences) and incubated for 1 min at room temperature (RT). The excess sample solution that was not attached to the grid was absorbed by the filter papers and removed. The sample was then fixed with a 4% paraformaldehyde (PFA; Biosesang) solution for 1 min at RT. After fixation, 2% of uranyl acetate was dropped onto the sample grids for 30 s for negative staining, and all the samples were dried overnight (O/N) at RT. Transmission electron microscopy (TEM) imaging was performed using a Tecnai F20 G2 transmission electron microscope (TEI).

### Western blotting analysis

The mExo and its modified/loaded forms were quantified using a Pierce BCA protein assay kit (Thermo Fisher). Exosomes (30 µg) were mixed with DW and 5X SDS loading buffer (250 mM Tris-HCl, 0.5 M DTT, 10% SDS, 0.25% bromophenol blue, and 50% glycerol) and heated at 98.5 ℃ for 10 min. Exosomal proteins were separated by 10% SDS-PAGE and transferred to a nitrocellulose membrane using a trans-blot turbo transfer system (Bio-Rad). The transferred membrane was blocked using 3% bovine serum albumin (BSA) and 0.3% skim-milk-containing TBST at RT for 1 h. The blocked membrane was incubated with primary TSG101 (1:1000 in 1% BSA-containing TBST; Abcam, ab83), CD9 (1:1000 in 1% BSA-containing TBST; Novus Biologicals, NU500-494), calnexin (1:1000 in 1% BSA-containing TBST; Abcam, ab227310), casein (1:2000 in 1% BSA-containing TBST; Abcam, ab166596), and MFG-E8 (1:1000 in 1% BSA-containing TBST; R&D systems, AF2805) antibodies at 4 ℃ for O/N. Membranes incubated with each primary antibody were washed using TBST and incubated with HRP-conjugated secondary antibody (1:1000 in TBST; GeneTex, GTX213111-01, and GTX231110-01 for anti-mouse and rabbit, respectively, and Abcam, ab6741 for anti-goat) at RT for 1 h. Finally, electrochemiluminescent substrate solutions (Bio-Rad) were poured onto the membrane for 1 min, and chemiluminescence signals were detected using a ChemiDoc instrument (Thermo Fisher).

### Surface modification of mExo by post-insertion

All surface-modified mExo used in this study were formulated using the following optimized post-insertion methods. First, 500 µg of mExo were mixed with 16:0 PE lipid with functional moieties, dissolved in dimethyl sulfoxide (DMSO), with a final DMSO of 5%, at *w/w* ratios of 20:1 and 10:1 (mExo:lipid-conjugated functional moieties), and the incubation was held for 2 h at 40 ℃. After incubation, the modified mExo was subjected to an ultrafiltration step (12,000 x g, 5 min at RT) to remove uninserted free lipids using a 100 K MWCO Amicon Ultra-0.5 centrifugal filter (Merck). Additionally, the same post-insertion procedure (incubation at 40 °C for 2 h) was also applied to intact mExo. To confirm whether the post-insertion step was successful, three different types of validation were conducted.

The modified mExo with 1,2-dipalmitoyl-sn-glycero-3-phosphoethanolamine-N-(biotinyl) (PE-biotin) was reacted with 50 µg of streptavidin-Flamma 496 (Stv-F496, in PBS, Bioacts) for 15 min at RT. Then, 25/50 µg of mExo-biotin/Stv-F496 was loaded onto a 10% non-SDS native polyacrylamide gel, and electrophoresis was conducted at 180 V for 40 min. The mExo-biotin/Stv-F496 complex within the gel was detected using a real-time whole-body in vivo/in vitro imaging system (IVIS) Lumina instrument (PerkinElmer).

To confirm the post-insertion further, mExo were incubated with 1,2-dipalmitoyl-sn-glycero-3-phosphoethanolamine-N-dibenzocyclooctyl (PE-DBCO) at a 10:1 (mExo:PE-DBCO) ratio (w/w). Next, 2 mM of Flamma 675-conjugated azide (Az-F675, in DMSO, Bioacts) was added to induce a click reaction with DBCO displayed on the mExo membrane. After 30-min incubation at 37 ℃, 1.5 × 10^5^ HEK293T cells seeded on 35 pi glass-bottom confocal dishes (SPL) were treated with 100 µg/mL of mExo-DBCO/Az-F675 complexes were treated to with 100 µg/mL of mExo-DBCO/Az-F675 complexes for confocal fluorescence imaging. After 2 h of incubation, all cells were washed three times with prewarmed Dulbecco’s phosphate buffered saline (DPBS) for 5 min, and fixed with 4% PFA for 10 min at RT. The fixed cells were washed with DPBS, and incubated with Hoechst 33,342 solution (Invitrogen) for 7 min. After staining, all samples were subjected to DBPS washing, and filled with 2 mL of DPBS to prevent the sample from drying. The prepared samples were imaged using a confocal microscope (Leica TCS SP5, Leica). Correlation analysis of the fluorescence signals was performed using LAS X and Prism 8.0 software.

Surface modification of mExo can be directly confirmed using a UV-vis spectrophotometer. The mExo surface was modified with 1,2-dipalmitoyl-sn-glycero-3-phosphoethanolamine-N-(6-((folate)amino) hexanoyl) (folate cap-PE, PE-FA; Avanti Polar Lipids). The 250 µg of mExo-FA complexes were loaded in transparent cuvettes, and the presence of FA inserted into the mExo membrane was confirmed by UV-vis spectra analysis at 200‒800 nm.

### Evaluating serum stability of mExo-FA

To evaluate the stability of mExo-FA in physiological conditions, F675-labeled mExo-FA were incubated in mouse serum for 24 h at 37 ℃. Mouse serum was isolated according to a serum preparation protocol provided by Thermo Fisher. After 24 h of incubation, the dissociated fluorescence dye (F675) and FA from mExo-FA were removed by 100 K MWCO filtration. Then, the remnant dye and FA on the mExo membrane were analyzed by using UV-vis spectra analysis.

### Loading of doxorubicin in mExo

For in vitro studies, 50 µg of Dox-HCl (10 µg/µL stocked in DMSO; FutureChem) was incubated with 200 µg of mExo/mExo-FA (total volume: 200 µL with PBS) at 4 ℃ O/N using the programmable rotator. After incubation, Dox-loaded mExo species were subjected to ultrafiltration to remove unloaded free Dox from the samples using a 100 K MWCO Amicon Ultra-0.5 centrifugal filter. For in vivo studies, 100 µg of Dox-HCl was loaded into 200 µg of mExo using the same protocol as for in vitro studies. Before using Dox@mExo/mExo-FA, loaded Dox was quantified by measuring the absorbance at 480 nm using a SpectroMax Microplate Reader (Molecular Devices). To calculate the loading capacity of Dox in mExo, the standard curve was set by measuring serially diluted Dox (from 50 µg/200 µL to 1.56 µg/200 µL). The loaded Dox was measured, and the amount of Dox was calculated based on the standard equation. The loading capacity of Dox was calculated using the following equation;

$${\text{Loading}}\,{\text{capacity}}\left( \% \right) = \frac{{\left( {Do{x_{total}}\left( {\mu g} \right) - Do{x_{unloaded}}\left( {\mu g} \right)} \right)}}{{Dox@mExo\left( {\mu g} \right)}}x100$$

### Release profile of dox loaded in mExo

After Dox loading, 100 µL of Dox-loaded mExo-FA were placed into a 10 K MWCO Slide-A-Lyzer dialysis device (Thermo Fisher). Each dialysis unit was inserted into 1.5-mL microcentrifuge tubes filled with acidic PBS (pH 6.8) and neutral PBS (pH 7.4). The tubes installed with dialysis devices were placed in a programmable thermomixer (KBT) and incubated at 37 ℃ with gentle shaking (600 rpm) for various time points. The concentration of released Dox was determined by measuring the absorbance at 480 nm using a UV-vis spectrophotometer.

### Cell culture

HEK293T cells were maintained in Dulbecco’s modified Eagle’s medium (DMEM, Hyclone) supplemented with 10% FBS (Atlas) and 1% antibiotics-antimycotic (Gibco). A549 (human lung cancer cell line, ATCC) and HCT116 (human colorectal cancer cell line, ATCC) cells were cultured in RPMI1640 (Welgene) with 10% FBS and 1% antibiotic-antimycotic solution. All cells were incubated at 37 ℃ with 5% CO_2_.

### Surface binding analysis of mExo/mExo-FA

A549 and HCT116 cells were seeded in a 60-mm cell culture dish (SPL) at a density of 3.0 × 10^5^ cells per dish, and incubated for 24 h. All cells were treated with 50 µg/mL Flamma 675-labeled mExo/mExo-FA and incubated at 4 ℃ for 30 min. After incubation, all cells were washed three times with ice-cold PBS, and samples for confocal fluorescence imaging were prepared using the same protocol aforementioned.

### Receptor-mediated cellular uptake of mExo/mExo-FA

To visualize the uptake level of mExo/mExo-FA, HCT116 cells were seeded in a 35-mm glass-bottom confocal dish (SPL) at a density of 2.0 × 10^5^ cells per dish and incubated with 50 µg/mL of mExo/mExo-FA in the presence or absence of anti-FRα antibody (1:500 diluted in cell culture medium, Thermo Fisher, MA5-23917). After 1.5 h incubation, all samples for confocal fluorescence imaging were prepared using the same protocol aforementioned. All confocal images were initially converted to an 8-bit format using Image J software. Subsequently, a Region of Interest (ROI) was defined within the intracellular region, excluding the cell surface, utilizing a freehand selection tool. Quantitative analysis was then performed by measuring the integrated density within the designated ROI. Prior to conducting statistical analysis, we confirmed that there were no significant differences in the ROI area for each group.

### In vitro cytotoxicity of mExo species

A549 and HCT116 cells (5.0 × 10^3^ cells) were placed in a 96-well cell culture plate (SPL). To check the cytotoxicity of mExo and mExo-FA, two distinct cell lines were treated O/N with various concentrations of mExo/mExo-FA (12.5, 25, 50, 100, and 200 µg/mL). After treatment, the cell culture medium was replaced with a medium containing a one-tenth volume of Cell Counting Kit-8 (CCK8, Dojindo) solution. After 1 h incubation, the absorbance was measured using a microplate reader at a wavelength of 450 nm. To evaluate the in vitro cytotoxicity of Dox@mExo-FA, the same density of HCT116 was seeded in a 96-well culture plate. HCT116 was treated with free Dox and Dox@mExo-FA (based on Dox concentrations of 1, 10, 25, and 50 µg/mL) and incubated for 12 h. The following CCK8 analysis progressed using the aforementioned protocol.

### In vivo biodistribution analysis

All animal experiments were performed in accordance with the International Guide for the Care and Use of Laboratory Animals and were approved by the Korea Institute of Science and Technology. Immunodeficient BALB/c nude mice (CAnN.Cg-*Foxn1*^*nu*^/Crl, n = 5 per each time point group) were used to monitor the in vivo biodistribution of mExo-FA. To establish HCT116 tumor-bearing mice, 5 × 10^6^/100 µL HCT116 cells (in RPMI1640) were subcutaneously injected into the left flanks of nude mice. When the implanted tumor size reached 200 mm^3^, 200 µg of Flamma 675-labeled mExo/mExo-FA (dispersed in 100 µL of PBS) were administered by intravenous (IV) injection and whole-body fluorescence signals of mExo species were tracked at various time points (2, 4, 6, 8, 12, and 24 h) using the IVIS instrument. After the whole-body biodistribution was checked, all mice were anesthetized using 2.5% isoflurane gas and dissected to extract their major organs (liver, lung, spleen, heart, and kidney) and tumors for ex vivo fluorescence imaging. The remaining fluorescence signals in the organs and tumors were also analyzed using the IVIS Lumina equipment. To confirm FA-mediated tumor targeting at the early time point (2 h), mExo/mExo-FA-injected tumor-bearing mice were sacrificed to collect tumors. After tumor collection, all samples were fixed in 4% PFA O/N. All fixed tumors were frozen using the optimal cutting temperature (OCT) compound (SAKURA). OCT-embedded tumors were cryo-sectioned (10-µm-thick) using a Microm HM525 NX Cryostat (Thermo Fisher). All premounted sections were incubated with Hoechst 33,342 solution for 10 min and covered with mounting medium (Thermo Fisher). The tumor volume was calculated using the formula; (width^2^ × length)/2.

### In vivo therapeutic effect of Dox@mExo-FA on tumor growth

To evaluate the therapeutic effect of Dox@mExo-FA, all controls and mExo species (PBS, free Dox, mExo-FA, and Dox@mExo-FA; an equivalent dose of 3 mg/kg of Dox dispersed in 100 µL of PBS) were injected intravenously into HCT116 tumor-bearing mice when the average tumor size reached approximately 50‒70 mm^3^. The five-repeated injections were performed every three days, and tumor sizes and body weights were measured every day. All subjects were sacrificed three days after the final injection, and tumor tissues were collected for measuring tumor weight and histological analysis.

### Histological analysis after treatment of Dox@mExo-FA

Dissected tumor tissues were fixed with 4% PFA for 24 h. After fixation, all fixed tissues were subjected to dehydration and paraffin embedding. Paraffin-embedded tumor tissues were sectioned at 6-µm thickness, and a TUNEL assay (Promega) was conducted to confirm cancer cell apoptosis according to the manufacturer’s instructions. TUNEL-positive cells were confirmed by using a confocal microscope, and their fluorescence intensity was analyzed using Fiji ImageJ software.

### Systemic toxicity of Dox@mExo-FA

To confirm the systemic toxicity of Dox@mExo-FA, whole blood, spleen, and heart were collected from tumor-bearing mice administered with PBS and each Dox-related group (free Dox, mExo-FA, and Dox@mExo-FA). To assess liver damage by Dox, aspartate aminotransferase (AST) and alanine aminotransferase (ALT) levels were analyzed by DKKorea, a nonclinical contract research organization institution. In addition, heart tissues were used for hematoxylin and eosin (H&E) staining to verify the well-known cardiotoxicity of Dox [22]. H&E staining was performed according to the same protocol used in a previous study [23].

### Statistical analysis

A statistical analysis was performed using Prism 8.0 (GraphPad). Statistical significance was determined using one-way or two-way analysis of variance (ANOVA) with Tukey’s *post-hoc* test and two-tailed t-test. A *P*-value less than 0.05 was considered statistically significant.

## Results

### Surface functionalization of mExo by post-insertion

**Fig. 1:**
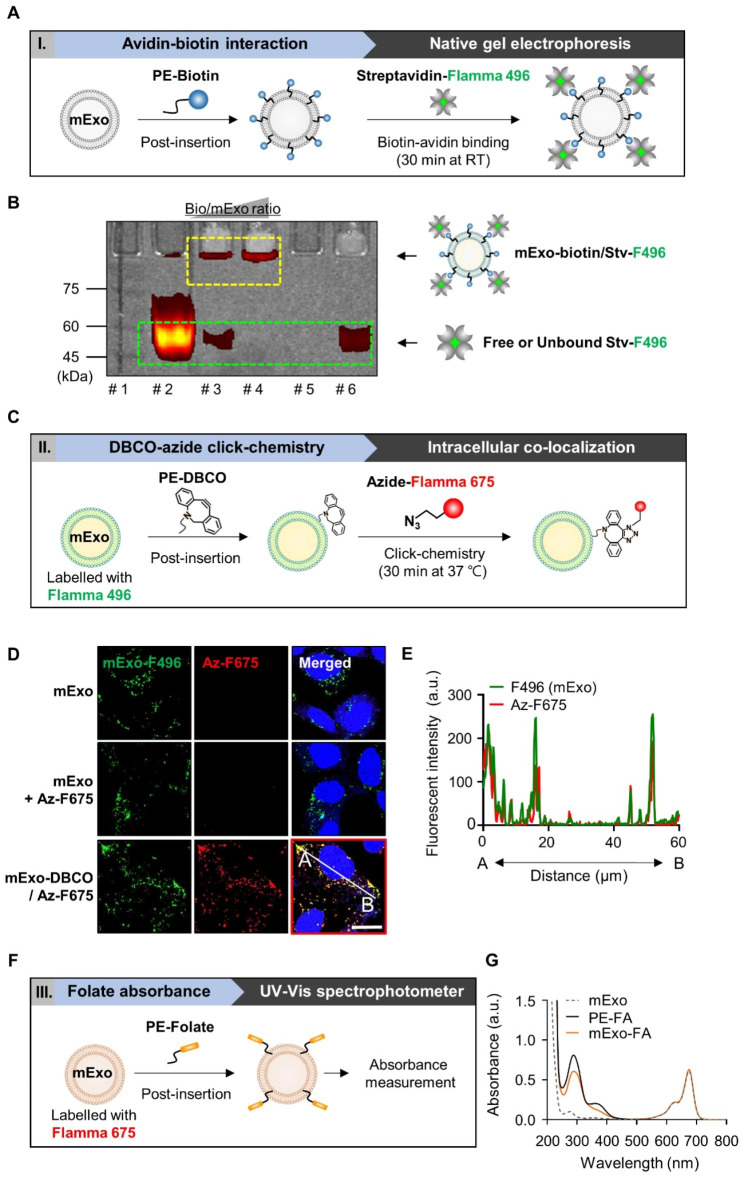
Validation of optimized surface modification of mExo. **(A)** Schematic illustration showing validation strategy using avidin–biotin interaction. **(B)** Native-PAGE gel image for confirming mExo-biotin/Stv-F496 complex. Each lane indicates following groups; lane 1: size marker; lane 2: free Stv-F496; lane 3: mExo-biotin/Stv-F496 complex with 20:1 mExo/bio weight ratio; lane 4: mExo-biotin/Stv-F496 complex with 10:1 mExo/bio weight ratio; lane 5: mExo only; and lane 6: mExo + Stv-F496 (no biotin insertion). The box with a yellow dashed line shows the complexes of mExo-biotin/Stv-F496, and the green dashed line indicates unbound free Stv-F496. **(C)** Schematic illustration of click chemistry-based validation strategy. **(D)** Confocal fluorescence images of visualizing colocalization of F496-labeled mExo (green) and azide-F675 (red). Scale bar: 50 μm. **(E)** Analysis of the correlation of colocalized fluorescence signals between the A-B line in the red box in Fig. 1D. The Pearson correlation coefficient was calculated using GraphPad Prism 8.0. **(F)** Schematic illustration indicating validation measuring the absorbance of FA. **(G)** UV-vis spectra of FA-modified mExo. The absorbance peaks at approximately 680 and 290 nm indicate mExo (F675) and PE-FA, respectively

Stable insertion of functionalized PE-lipid chains into mExo membranes was achieved using a post-insertion method. To determine whether lipid-conjugated functional residues were successfully inserted into the mExo membrane, three distinct biochemical analyses were performed based on (1) avidin‒biotin interaction, (2) click chemistry, and (3) FA absorbance (Fig. 1).

Given that the avidin‒biotin interaction is one of the strongest biological interactions, exhibiting a markedly low dissociation constant (K_d_ = 1.3 × 10^− 15^ M at pH 5.0) [24], post-insertion of lipid-conjugated biotin (16:0 biotin-PE) into the mExo membrane could be easily detected by incubation with Flamma 496-conjugated streptavidin (Stv-F496) (Fig. 1A). As shown in Fig. 1B, free or unbound Stv-F496 signals were detected at approximately 53 kDa (green box), whereas Stv-F496 signals, which corresponded to the avidin‒biotin interaction (yellow box, mExo-biotin/Stv-F496), were upshifted. In particular, free Stv-F496 signals were detected with a 20:1 (mExo:biotin) weight ratio, while most Stv-F496 was bound to PE‒biotin incorporated into mExo at a 10:1 (mExo:biotin) weight ratio. Considering these results, we employed an mExo:PE‒biotin (*w*/*w*) ratio of 10:1 for the post-insertion-induced functionalization of mExo in all subsequent experiments.

Next, lipid-conjugated DBCO (16:0 PE-DBCO) and Flamma 675-labeled azide (Az-F675) were used to validate the post-insertion method. DBCO and azide are frequently employed in biological cross-linking reactions as reactive groups that drive Cu-free click chemistry [25]. After the post-insertion of PE-DBCO into the F496-labeled mExo (green signal) membranes, DBCO-displaying mExo were incubated with Az-F675 (red signal) to achieve cross-linking of the DBCO and azide groups (Fig. 1C). The colocalization of the two fluorescence signals of F496 and F675 was confirmed by fluorescence confocal imaging following the treatment of HEK293T cells with mExo and modified mExo. As depicted in Fig. 1D-E, the fully assembled group (mExo-DBCO/Az-F675) exhibited highly colocalized fluorescence signals (Pearson correlation coefficient: 0.9) of mExo (labeled with F496) and Az-F675. Az-F675 signals were not detected, even after incubation with the azide group in mExo, which was not pre-inserted into the PE-DBCO group (mExo + Az-F675). These results confirmed that the optimized post-insertion conditions made it possible to insert PE-conjugated moieties into the mExo membrane.

Finally, the surface functionalization of mExo was directly assessed by spectrophotometric analysis (Fig. 1F). According to a previous study, 282.5 nm is the maximum absorption wavelength for detecting FA in pharmaceutical formulations (λ_max_) [26]. However, the lipid-conjugated FA (16:0 PE-FA) utilized in this study displayed a bathochromic shift, resulting in an absorption peak at 290 nm. Based on speculation that FA inserted into the mExo membrane would retain its intrinsic physicochemical properties, 16:0 PE-FA was inserted into F675-labeled mExo to confirm the absorbance peaks at 290 and 680 nm for FA and mExo, respectively. As shown in Fig. 1G, the mExo-FA group showed two absorbance peaks at approximately 680 and 290 nm, whereas the nonfunctionalized mExo group exhibited a single absorbance peak at 680 nm (Fig. 1G). Next, the post-insertion efficiency of FA into mExo was calculated from their respective concentrations using the standard curve of the PE-FA absorbance spectra. The calculated insertion efficiency was 71.3% (Fig. S2A). Moreover, it was confirmed that FA functionalization of the mExo membrane was stably maintained in serum (Fig. S2B). These results confirm the successful insertion of PE-FA into the mExo surface membrane.

### Characterization of mExo-FA

**Fig. 2:**
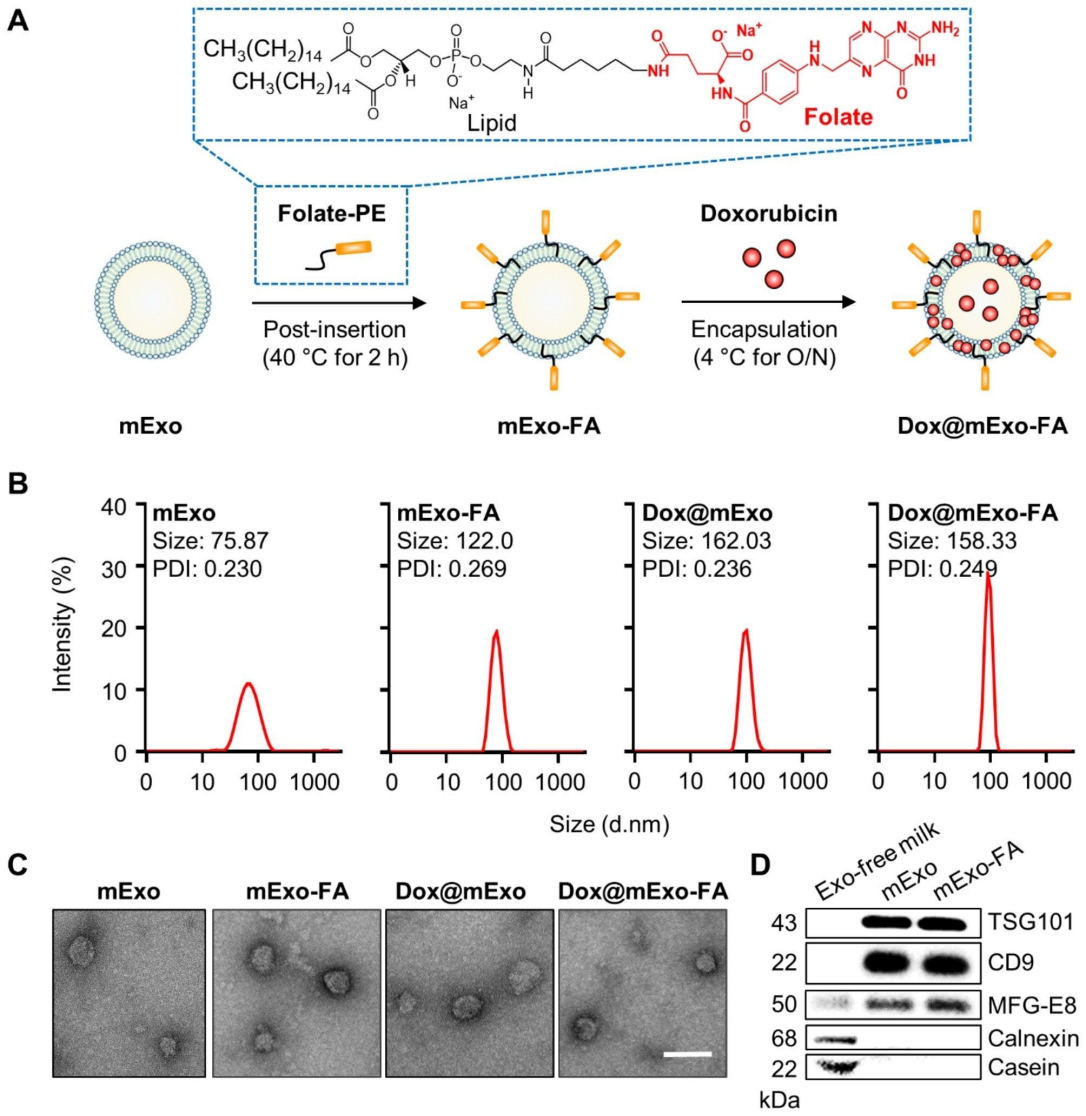
Characterization of the surface functionalized mExo with FA. **(A)** Schematic illustration showing a process of surface engineering and Dox loading. The box with a blue dashed line demonstrates the modified chemical structure of 16:0 PE-FA obtained from Avanti Polar Lipids. **(B)** Size distribution of mExo species (mExo, mExo-FA, Dox@mExo, and Dox@mExo-FA). **(C)** TEM images of mExo, mExo-FA, Dox@mExo, and Dox@mExo-FA. Scale bar: 100 nm. **(D)** Western blotting analysis of TSG101, CD9, MFG-E8, calnexin, and casein. TSG101 and CD9, representative positive exosome markers; MFG-E8, milk-exosomal proteins; calnexin, negative marker of exosome; casein, representative contaminants from milk

To apply engineered mExo for targeted cancer therapy, milk-derived exosomes were designed that express FA on their surface and load Dox (Dox@mExo-FA) by an optimized post-insertion technique (Fig. 2A). Intact mExo and the modified species (mExo-FA, Dox@mExo, and Dox@mExo-FA) were characterized by DLS, TEM imaging, and western blotting analysis. The Z-average and PDI values from DLS revealed that functionalized mExo (mExo-FA and Dox@mExo-FA) had particles with low polydispersity and average diameters of 122.0 and 158.33 nm, respectively. These results imply that the process of FA insertion and Dox loading may slightly increase the hydrodynamic size of mExo. TEM imaging was then used to determine whether the morphology of the modified mExo (mExo, mExo-FA, Dox@mExo, and Dox@mExo-FA) was maintained after surface functionalization and Dox loading. As depicted in Fig. 2C, mExo-FA and Dox@mExo-FA exhibited spherical shapes similar to those of unmodified mExo. Based on western blotting analysis, both mExo species (mExo and mExo-FA) were positive for exosome-related markers (TSG101 and CD9) and milk fat globule EGF factor 8 (MFG-E8), a major protein in mExo. However, calnexin (endoplasmic reticulum marker) and casein, a major milk protein contaminant, were not detected in mExo (Fig. 2D). In addition, the size exclusion chromatography data showed only one peak, indicating the high homogeneity of mExo (Fig. S3A), supporting the conclusion that the purification steps in this study were sufficient to remove protein contaminants from milk.

### Receptor-mediated cellular uptake of mExo-FA

Next, an attempt was made to determine whether surface functionalization with FA could improve targeted mExo delivery. Here, HCT116 cancer cells that highly express FR on their surfaces [27] and A549, a representative cell line that exhibits low levels of FR expression [28], were employed. The immunostaining results revealed comparatively weak red fluorescence signals in A549 cells and strong FR fluorescence signals in HCT116 cells (Fig. S4A). To examine the cytotoxicity of mExo and mExo-FA, A549 and HCT116 cells were treated with various concentrations of mExo and mExo-FA (12.5, 25, 50, 100, and 200 µg/mL). We found that neither mExo nor mExo-FA affected cell viability (Fig. S4B).

**Fig. 3:**
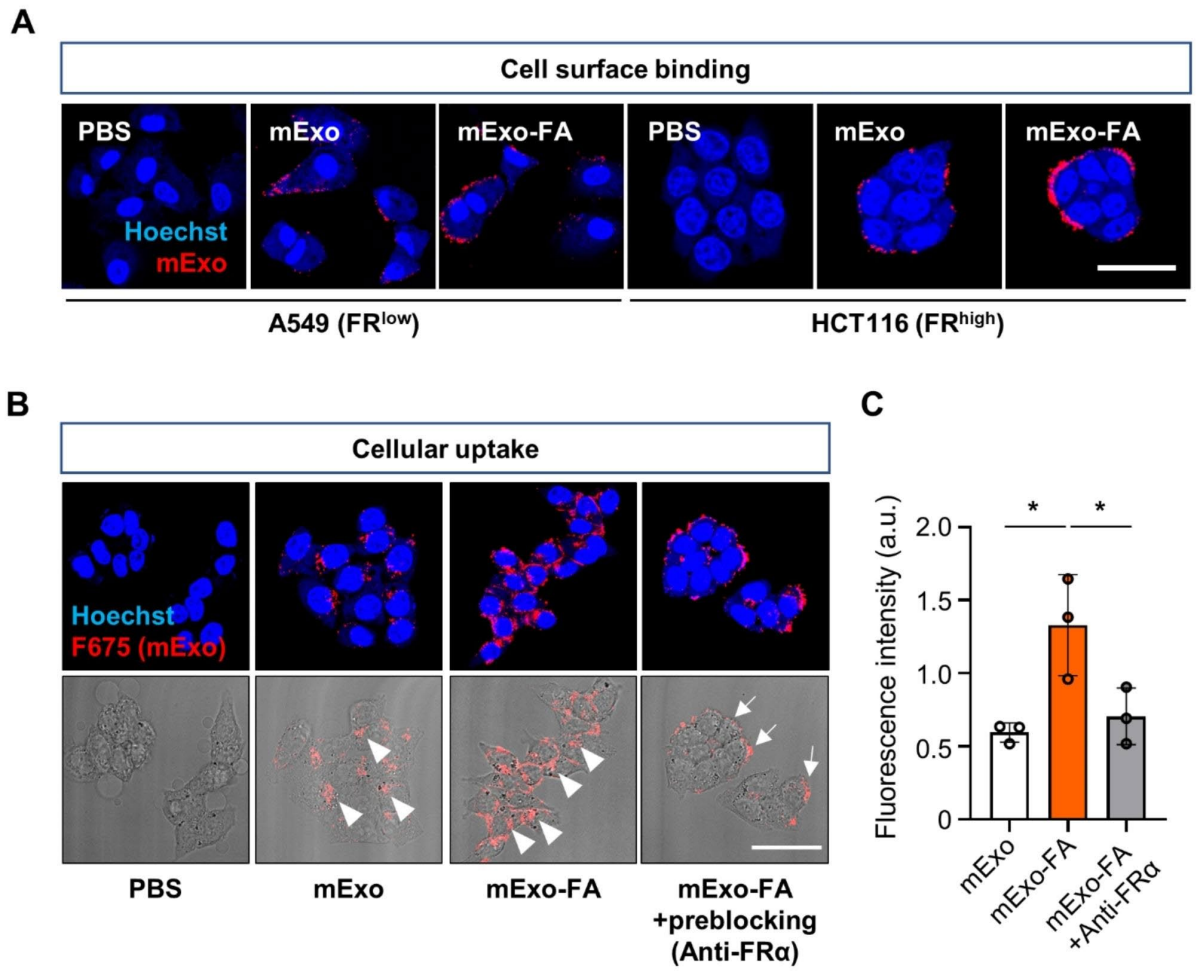
Receptor-mediated cellular uptake of mExo-FA. **(A)** Representative fluorescence images indicating binding of mExo and mExo-FA on the surfaces of both cells. Scale bar: 50 μm. **(B)** Representative fluorescence confocal and bright-field images showing cellular uptake of 50 µg/mL mExo/mExo-FA in HCT116 with FR preblocking with anti-FRα (1:500 *v/v* ratio). Arrowheads indicate intracellular uptake of mExo/mExo-FA in HCT116 cells. White arrows indicate mExo/mExo-FA stacked (not intracellularly uptaken) on cell surface. **(C)** Quantified graph indicating intracellular uptake level of mExo/mExo-FA. One-way ANOVA followed by Tukey’s multiple comparisons was performed: *P < 0.05, ***P < 0.001

To determine whether FA functionalization of mExo improves cellular uptake through FR-mediated interactions, A549 (FR^low^) and HCT116 (FR^high^) cancer cells were treated with mExo or mExo-FA (50 µg/mL). As shown in Fig. 3A, the fluorescence imaging results revealed a notable increase in mExo-positive red fluorescence signals, which is exclusively evident in the group of HCT116 cells treated with mExo-FA. This finding suggests that surface-inserted FA promoted targeted binding to FR^high^-cell lines.

Consistent with the results of the cell-binding analysis, the cellular uptake of mExo-FA in HCT116 cells was significantly higher than that of mExo treatment. To visualize the FA-dependent cellular uptake of mExo-FA, the distributions of fluorescence-labeled mExo and mExo-FA in HCT116 cells were analyzed. Compared with the unmodified mExo- and anti-FRα-treated groups, the mExo-FA-treated group showed remarkably enhanced intracellular fluorescence signals (Fig. 3B-C). In particular, F675-labeled mExo-FA were detected in the cytoplasm of HCT116 cells, whereas, in the anti-FRα pretreatment group, most fluorescent mExo-FA signals were detected on the cell surface rather than within cells. This preblocking of FR made FA-independent cellular uptake of mExo-FA possible at levels comparable to those of intact mExo. This finding suggests that blocking FR with an anti-FR antibody may interfere with the internalization of mExo-FA by interrupting the interaction between FR, expressed on HCT116 cells, and FA, inserted in the mExo membranes.

### mExo-mediated dox delivery into FR^high^ HCT116 cells

**Fig. 4:**
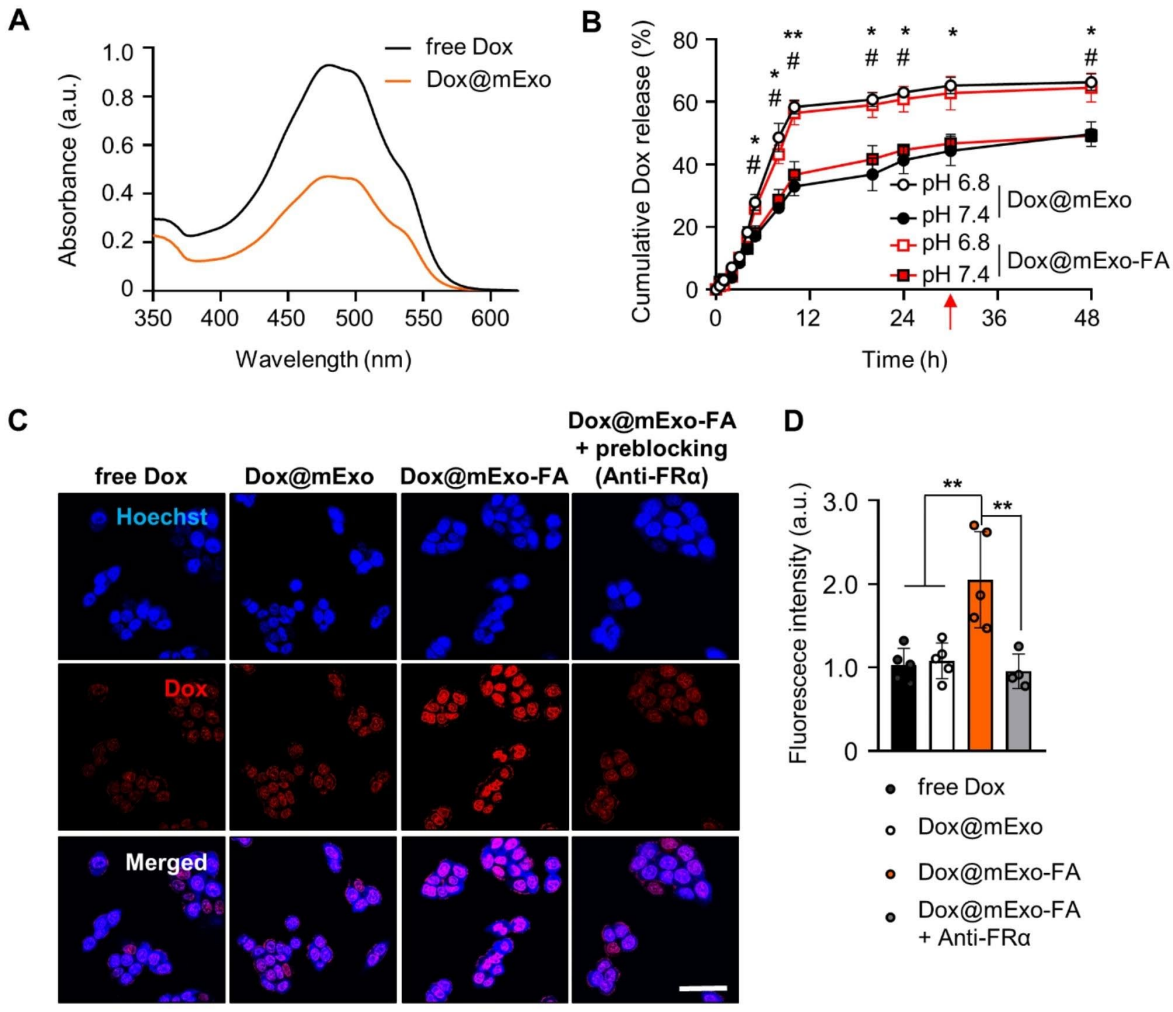
Dox loading and mExo-FA-mediated Dox delivery into HCT116 cells. **(A)** UV-vis spectra absorbance curves demonstrating the loading capacity of Dox (black line: absorbance of 50 µg Dox; orange line: absorbance of Dox loaded into mExo). **(B)** Time-dependent cumulative Dox release from mExo-FA and mExo at pH 6.8 and pH 7.4. The release profile of Dox was measured at various time points (10 and 30 min, and 1–5, 8, 10, 20, 24, 30, and 48 h). The red arrow indicates the time point (30 h) of arrival of plateau of Dox release at pH 6.8. (Plateau of Dox release at pH 6.8: 64.6%; at pH 7.4: 50.7%, mExo at pH 6.8: 66.3%; at pH 7.4: 49.2%). * and # indicate statistical significance between pH 6.8 and pH 7.4 in each group (Dox@mExo / Dox@mExo-FA), respectively. **(C)** Confocal images showing cellular uptake of Dox by Dox@mExo-FA in HCT116 cells (based on 1 µg/mL of Dox concentration). Scale bar: 50 μm. **(D)** Quantified graph demonstrating intracellular fluorescence intensity of Dox from **(C)**. One-way ANOVA with Tukey’s multiple comparison test was conducted for every statistical analysis: *P < 0.05, ** *P* < 0.01

After validating the receptor-mediated cellular uptake of mExo-FA, the intracellular delivery of Dox into FR^high^ HCT116 cells using mExo-FA as a drug carrier was assessed. The loading capacity of Dox into mExo-FA was approximately 30–35% on average for each batch (Fig. 4A). Given the release behavior of mExo-FA-loaded Dox, Dox@mExo-FA exhibited increased Dox release by approximately 64.6% under weakly acidic (pH 6.8) conditions mimicking the tumor microenvironment (TME) when compared with that under neutral conditions (50.7%) at the same time point (Fig. 4B). A similar release profile was observed for Dox@mExo, indicating that FA functionalization by post-insertion did not affect Dox release (Fig. 4B). No significant differences in the mExo particle size were detected under acidic or neutral conditions (Fig. S5). Several studies have previously reported that mExo can maintain their stable membrane rigidity, even under acidic conditions [29, 30], whereas membranes of cell-derived exosomes are easily disrupted at a low pH. In addition, the solubility and protonation constant of Dox increase with the acidity of the environment [31–33]. Accordingly, Dox@mExo-FA can selectively improve Dox release in acidic tumor sites.

To determine whether FA functionalization of mExo can improve the uptake of Dox by cancer cells, HCT116 cells were treated with free Dox, Dox@mExo, and Dox@mExo-FA (1.0 µg/mL, based on Dox concentration) for 2 h. As a result, mExo-FA significantly enhanced the efficiency of Dox delivery in HCT116 cells (Fig. 4C-D). Consistent with the results in Fig. 3A, we observed that Dox@mExo/mExo-FA induced low levels of Dox uptake regardless of the presence or absence of FA (Fig. S6).

### Tumor-specific delivery of mExo-FA

**Fig. 5:**
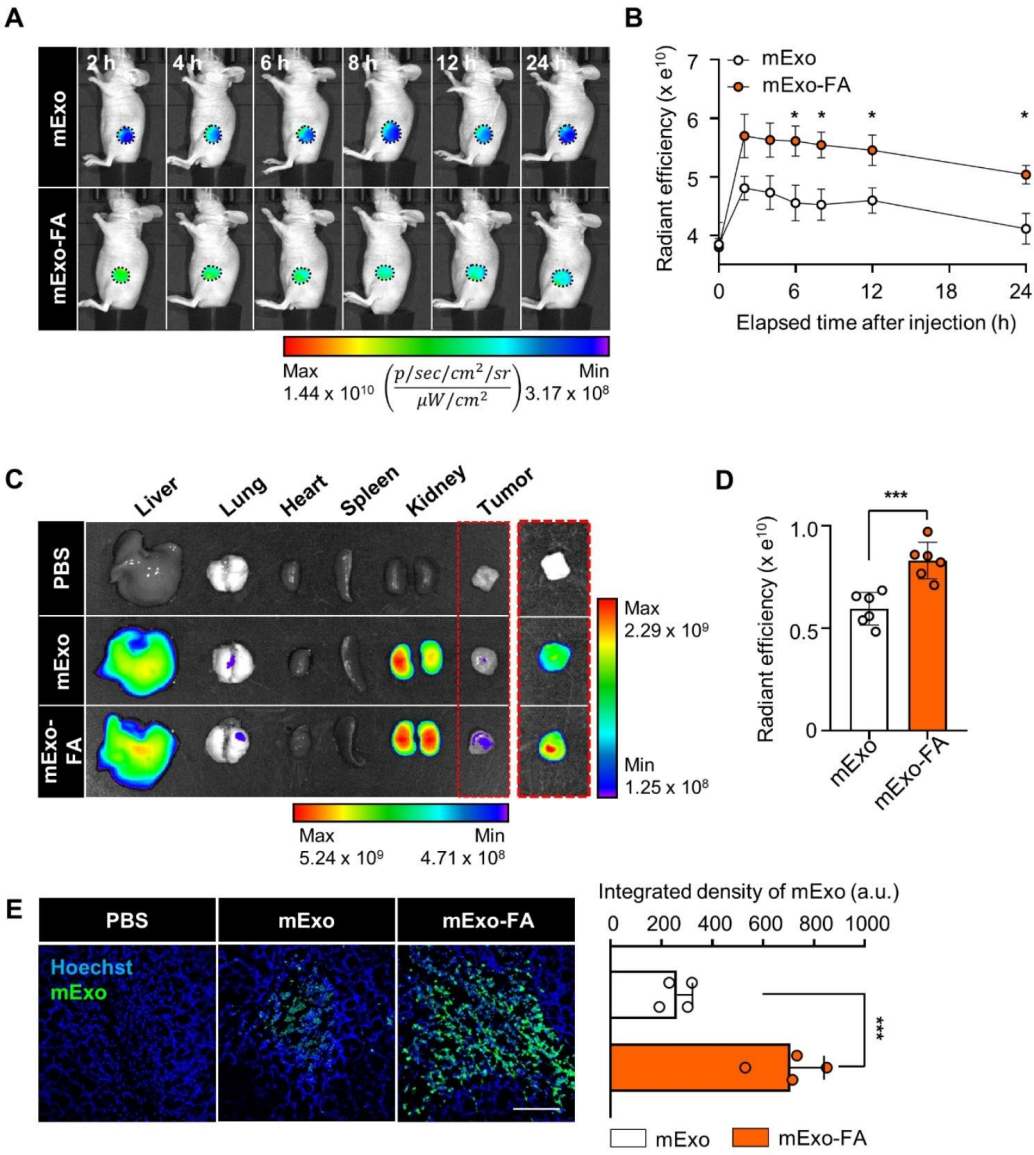
In vivo biodistribution of mExo-FA in HCT116 tumor-bearing mouse. **(A)** Representative fluorescence whole-body images of tumor-bearing mice at various time points (2, 4, 6, 8, 12, and 24 h after systemic 200 µg of mExo/mExo-FA injection). Displayed fluorescence signals were restricted to the tumor area demarcated by black dotted circles. Whole-body fluorescence images are presented in Fig. S7. The radiant efficiency in tumor tissue is calculated by ($\frac{p/sec/c{m}^{2}/sr}{\mu W/c{m}^{2}}$). **(B)** Quantified radiant efficiency of mExo/mExo-FA in primary tumor site (black dotted circle) of tumor-bearing mice in **(A)** at various time points (2, 4, 6, 8, 12, and 24 h after injection). **(C)** Ex vivo fluorescence image of major organs (liver, lung, heart, spleen, and kidney) and tumor tissue after 24 h of injection. **(D)** Quantified radiant efficiency of excised tumor tissues displayed in a red dot box in **(C)**. **(E)** Representative fluorescence images (left) and quantified fluorescence density graph (right) of F675-labeled mExo/mExo-FA in tumor tissue. Scale bar: 100 μm. The statistical significance was calculated using a two-tailed t-test; *P < 0.05, ***P < 0.001

Before the antitumor effects of Dox@mExo-FA were evaluated, the tumor accumulation efficiency of mExo-FA was examined. When the tumor size reached 200 mm^3^, mExo and mExo-FA (200 µg) were intravenously injected into the tail veins of HCT116 tumor-bearing mice, and the biodistribution of mExo-FA was monitored for 24 h. After 6 h of injection, it was observed that the fluorescence signals of mExo-FA were significantly higher than those of mExo (Fig. 5A-B, S7). The markedly increased intratumoral accumulation of mExo-FA was maintained for up to 24 h after injection. After whole-body fluorescence imaging was completed, all subjects were sacrificed and dissected to collect five major organs (liver, lung, spleen, heart, and kidney) and tumors. The fluorescence signals from organs and tumors revealed that mExo-FA remained at the tumor site for longer than unmodified mExo (Fig. 5C-D). As expected, mExo-FA was found to accumulate more than mExo in tumor tissues due to their targeting properties (Fig. 5E).

**Fig. 6:**
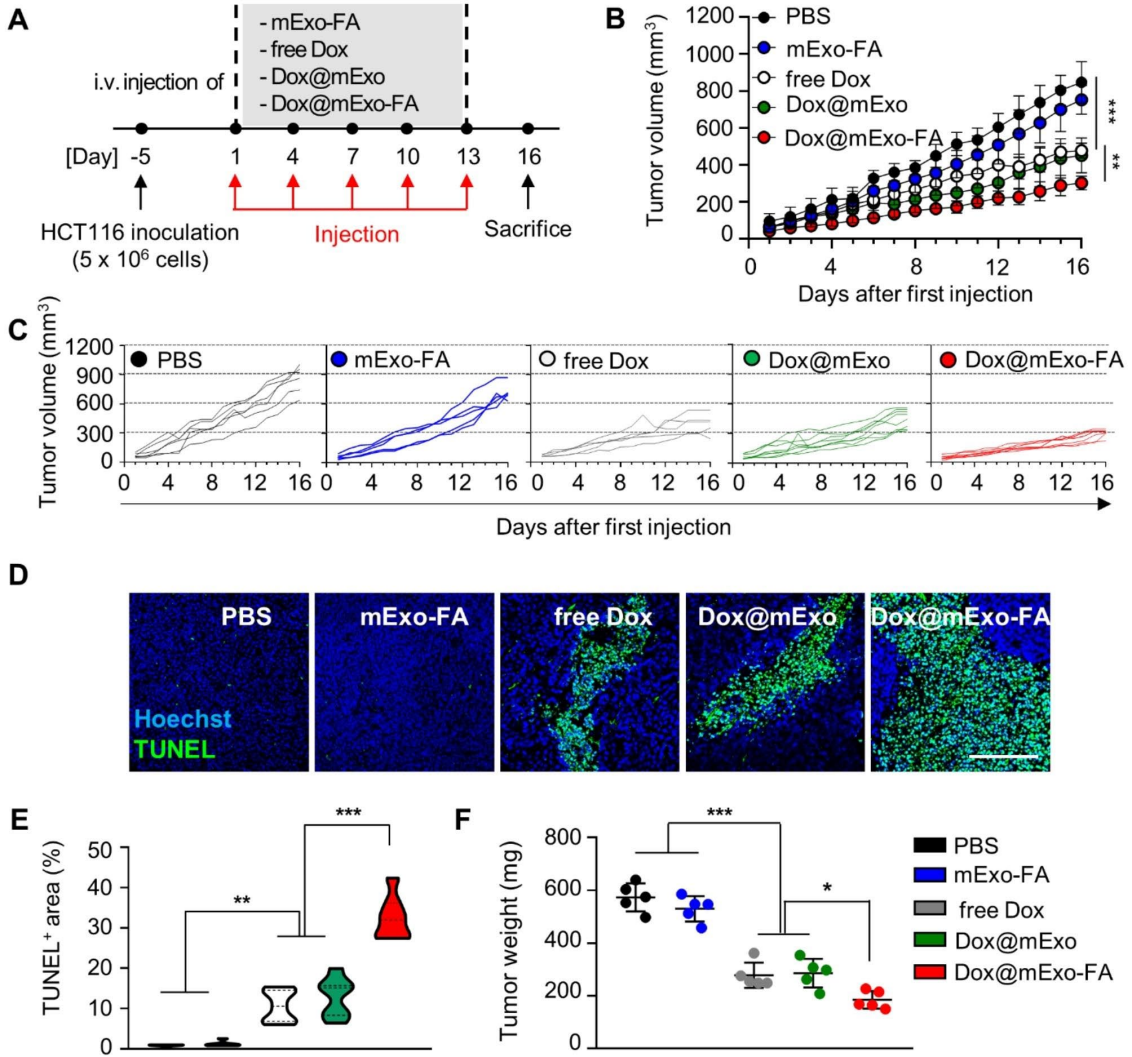
Therapeutic effect of Dox@mExo-FA in HCT116 tumor-bearing mouse. **(A)** Experimental scheme for assessing in vivo antitumor effect of Dox@mExo-FA. **(B-C)** Tumor growth curves for 16 days after injection of each group (n = 5–8 for each group; black circle: PBS; blue circle: mExo-FA; white circle: free Dox; green circle: Dox@mExo; red circle: Dox@mExo-FA). The administration dose was adjusted to 3 mg/kg of free Dox concentration. **(D-E)** A TUNEL assay for confirming apoptotic cells in tumor tissues three days after final treatment **(D)** and a quantified graph indicating a TUNEL-positive area **(E)**. Scale bar: 200 μm. (F) Tumor weight measured after three days of the last injection of all groups. One-way ANOVA with Tukey’s *post-hoc* multiple comparison was carried out for statistical analysis. n.s.: not significant, *P < 0.05, **P < 0.01, ***P < 0.001

### In vivo therapeutic effects of Dox@mExo-FA in HCT116-bearing mice and its in vivo toxicity

Next, the antitumor effects of Dox@mExo-FA in HCT116 tumor-bearing mice [34, 35] were evaluated. When the average size of HCT116 tumor xenografts implanted in the flank reached approximately 60 ± 10 mm^3^, mice were intravenously administered PBS, mExo-FA, free Dox, Dox@mExo, and Dox@mExo-FA at intervals of three days, at a dose equivalent to 3 mg/kg of Dox (Fig. 6A). As shown in Fig. 6B-C, treatment with Dox@mExo-FA significantly inhibited tumor growth. In contrast, treatment with PBS did not affect tumor progression (average tumor volume [mm^3^]; PBS, 853.6 ± 139.5; mExo-FA, 752.9 ± 69.8; free Dox, 477.6 ± 96.2; Dox@mExo: 451.1 ± 53.2; Dox@mExo-FA, 302.7 ± 16.5). In particular, the Dox@mExo-FA-treated group showed significant tumor growth inhibition with the little deviation between subjects, whereas the tumor growth inhibition patterns were various in the free Dox and Dox@mExo-treated groups (Fig. 6B-C). After all subjects were sacrificed, a TUNEL assay was performed to assess cancer cell apoptosis, and the tumors were weighed. Similar to the tumor volume measurement results, the Dox@mExo-FA-treated groups displayed a significantly increased TUNEL-positive area in the tumor tissues when compared with other groups (mExo-FA, free Dox, Dox@mExo-treated groups). This suggests that FA-functionalization of mExo can facilitate targeted Dox delivery to the tumor site. Consistent with the TUNEL assay results, Dox@mExo-FA markedly reduced the tumor weight (Fig. 6F).

**Fig. 7:**
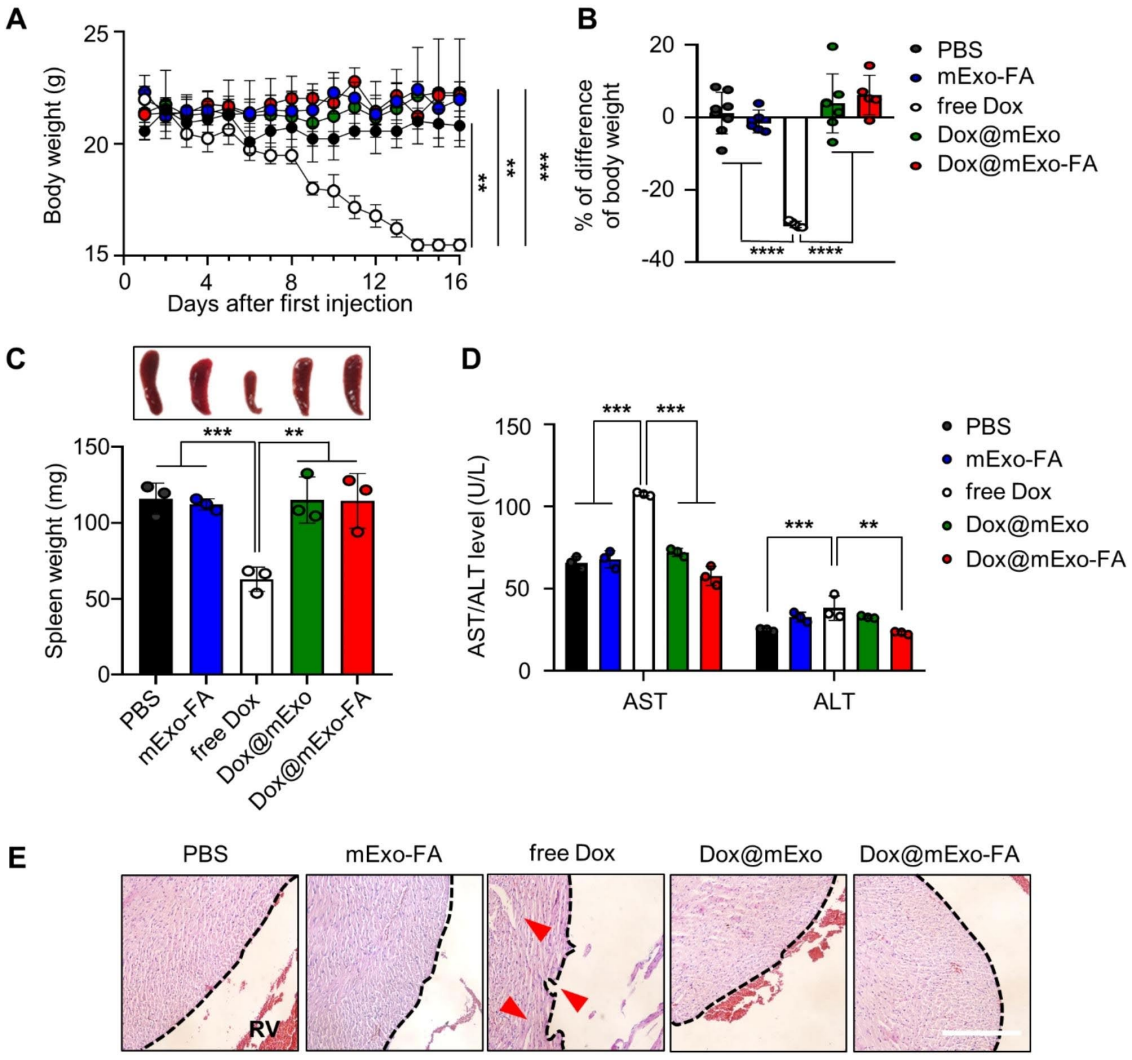
In vivo systemic toxicity of Dox@mExo-FA in HCT116 tumor-bearing mouse. **(A-B)** Body weight changes in mice during multiple administrations of PBS, mExo-FA, free Dox, Dox@mExo, and Dox@mExo-FA for 16 days (n = 5–8 for each group; black circle: PBS; blue circle: mExo-FA; white circle: free Dox; green circle: Dox@mExo; red circle: Dox@mExo-FA). **(C)** Spleen size and weight after five consecutive injections of PBS, mExo-FA, free Dox, Dox@mExo, and Dox@mExo-FA. **(D)** Blood analyses of mice treated with PBS, mExo-FA, free Dox, Dox@mExo, and Dox@mExo-FA for 16 days. **(E)** Representative H&E images of heart tissue from each group (black dashed line: tissue surface of heart; red arrows: damaged tissue structures; right ventricle (RV)). Scale bar: 100 μm. One-way ANOVA with Tukey’s *post-hoc* multiple comparisons was performed for statistical analysis: n.s: not significant, **P < 0.01, ***P < 0.001, ****P < 0.0001,

As depicted in Fig. 6B-C, free Dox-treatment also delayed tumor growth moderately; however, repeated systemic administration of free Dox resulted in notable body weight loss (Fig. 7A, average body weight [g]; PBS, 20.8 ± 1.3; mExo-FA, 21.98 ± 0.8; free Dox, 15.5 ± 0.5; Dox@mExo, 22.3 ± 2.1; Dox@mExo-FA, 22.1 ± 0.5). When the first body weight measurement was compared with the final one, the free Dox group displayed a weight loss of 29.5%, whereas the Dox@mExo-FA group showed a negligible difference in body weight (Fig. 7B). These results confirm that FA-functionalized mExo can induce tumor growth inhibition by delivering Dox without adverse effects. In addition to body weight loss, Dox has been reported to induce splenic contraction and severe cardiotoxicity [22, 36]. As expected, significantly reduced splenic size and weight (average spleen weight [mg]; PBS, 115.9 ± 8.39; mExo-FA, 112.2 ± 6.67; free Dox, 62.9 ± 6.5; Dox@mExo, 115.1 ± 12.4; Dox@mExo-FA, 114.5 ± 14.7) were observed in the free Dox-administered group (Fig. 7C). In addition to splenic shrinkage, blood analyses revealed significant changes in hematological parameters, such as AST and ALT, indicating free Dox-induced hepatic toxicity (Fig. 7D). H&E staining results also revealed that repeated systemic Dox administration caused significant histological damage in the heart tissue compared with the other treatment groups (Fig. 7E). Given that lethal systemic toxicity caused by Dox can be alleviated by loading it into mExo-FA, the results support the potential of developing surface-engineered mExo as a safe drug delivery system.

## Discussion

Exosomes have proved to be promising drug delivery vehicles, given their ability to deliver hydrophilic and hydrophobic therapeutic molecules with high biocompatibility [37]. Despite these advantages, isolating exosomes from cell culture media is time-consuming and labor-intensive. Indeed, the isolation of cell-derived exosomes can take up to a week while a series of processes are performed, from cell culture to exosome extraction. However, cell exosomes obtained from the aforementioned process afford very low yields, typically in microgram units per batch (obtained from approximately 400 mL of cell culture medium) [38]. Accordingly, scaled-up exosome manufacturing warrants the development of alternative sources to replace cell cultures for adequate production [5, 39]. Therefore, the emergence of milk-derived exosomes is expected to overcome the limitations of cell-derived exosomes as therapeutic drug carriers. In particular, the commercially available milk used in this study provides a relatively easy quality control source for exosome preparation compared with colostrum, which has a much higher protein and fat content.

The primary goal of a drug delivery system is to maximize the efficacy of drugs while minimizing their toxicity [40]. To achieve this, drugs must be delivered at a sufficient concentration to the desired regions. Therefore, milk exosomes should be modified with a functional moiety on their membranes for efficient tissue-specific delivery of various drugs. Instead of genetic modification at the cellular level, exosomes can be functionalized using a post-isolation modification strategy called ‘post-insertion’. Post-insertion facilitates the induction of functionality in lipid membrane-based nanomaterials such as liposomes [15, 41]. The post-insertion technique can decorate several exosomes using a relatively simple process without gene-related side effects, such as misinterpretation [14, 42]. For example, Gupta’s group previously reported a targeting system to deliver drugs, such as paclitaxel with colostrum-derived exosomes, using a chemical modification method, and activated ligands were covalently attached to the exosomal proteins [43, 44]. However, chemical modifications that mainly focus on amine-reactive crosslinker chemistry can affect the natural activity of exosomes by binding to the amino groups of functional proteins on the surface of exosomes. Furthermore, direct chemical modification methods may have limited applications because of their low site-selectivity compared with lipid-post-insertion methods, which are physical modifications that often result in the uncontrolled formation of covalent bonds between exosomes and ligands of interest [45, 46].

In this study, mExo with lipid-conjugated functional moieties were designed using the physical post-insertion method. Physical modifications, including approaches using liposome fusion or lipophilic molecules, require a high activation energy for successful insertion owing to their high phase transition temperature [47]. Although the reaction environment requiring relatively high temperatures can affect the activity of nanoparticles, the commercially available milk-derived exosomes used in this study retained their structure and function even under harsh conditions of physical modification (2 h at 40 °C incubation).

In addition, the lipid post-insertion method was used to improve insertion efficiency through high site selectivity and to secure versatility [45]. However, post-insertion methods based on passive hydrophobic interactions between mExo and lipid-conjugated ligands require accurate validation of membrane functionalization. As shown in Fig. 1, an attempt was made to determine whether post-insertion can be used to modify mExo appropriately through various biochemical analyses. Moreover, to optimize the precise post-insertion conditions for the functionalization of mExo, the mixing ratio of the mExo/lipid-conjugated functional moiety was controlled, ensuring that post-insertion was conducted successfully. As demonstrated in Fig. 3, we observed that FA-functionalization of mExo significantly enhances its capacity to bind to cancer cells expressing high levels of FR. Consequently, this leads to enhanced cellular internalization of mExo through receptor-mediated endocytosis, thereby resulting in improved targeted delivery of Dox to cancer cells. Despite the great drug delivery effects of mExo-FA, the administration of mExo to human subjects necessitates careful consideration of potential risks, particularly regarding cow milk allergy [48]. However, it’s worth noting that extensive research on mExo has established its high biocompatibility. Additionally, during the isolation process of mExo, steps can be taken to remove casein, a well-known allergen found in milk. Consequently, we have reasonable confidence that mExo retains significant promise as a drug delivery carrier. Therefore, providing mExo functionality by post-insertion would expand its potential of mExo for various therapeutic applications in preclinical and clinical studies.

## Conclusion

In this study, to confirm that the ability imparted by post-insertion works well, FA, a ligand of FR expressed in various cancer cells, was inserted into the mExo membrane. mExo-FA showed no significant toxicity in vitro and in vivo, and the functionalization of the mExo-membrane with PE-FA enhanced the tumor-targeting ability of mExo. In particular, FA-engineered mExo exhibited increased accumulation at the tumor site compared with the same quantity of intact mExo-injected group. After Dox was loaded into mExo-FA (Dox@mExo-FA), it was confirmed that mExo-FA can selectively deliver Dox to the tumor site and inhibit tumor growth without adverse effects including body weight loss, splenic contraction, hepatotoxicity, and cardiotoxicity. However, considering the complexity of the TME, multiple modifications may be necessary to improve the efficacy of drug delivery systems. One such modification involves addressing the extracellular matrix, a prominent constituent of the TME, which acts as a physical barrier impeding drug delivery to cancer cells [49]. Therefore, in addition to imparting tissue-targeting ability, it is conceivable to incorporate ECM-degrading enzymes onto the surface of mExo [50], which may contribute to the development of comprehensive anti-tumor strategies based on mExo.

Based on these findings, the optimized post-insertion technique offers an opportunity to use mExo for cancer drug delivery. Owing to their excellent safety, stability, biocompatibility, and mass productivity, they can be considered promising drug delivery agents that make oral administration possible. In this study, the surface functionalization of milk exosomes was verified using various methods. A milk exosome platform functionalized by post-insertion is expected to expand the potential of milk exosomes for various therapeutic applications in preclinical and clinical studies.

### Electronic supplementary material

Below is the link to the electronic supplementary material.

Supplementary Material 1

## Data Availability

All presented data in this manuscript and supporting information are available from the corresponding authors upon reasonable request.

## List of Abbreviations

mExo Milk: Derived exosomes
PE: Phosphatidylethanolamine
FA: Folate
DBCO: Dibenzocyclooctyne
Dox: Doxorubicin
FR: Folate receptor
PBS: Phosphate-buffered saline
DW: Distilled water
DLS: Dynamic light scattering
PDI: Polydispersity index
PFA: Paraformaldehyde
RT: Room temperature
O/N: Overnight
TEM: transmission electron microscopy
BSA: Bovine serum albumin
DMSO: Dimethyl sulfoxide
MWCO: Molecular weight cut-off
IVIS: In vivo/in vitro imaging system
AST: Aspartate aminotransferase
ALT: Alanine aminostransferase
H&E: Hematoxylin and eosin
TME: Tumor microenvironment
