# Supplementary material for: Post-insertion technique to introduce targeting moieties in milk exosomes for targeted drug delivery
Source: Biomater Res. 2023 Nov 29;27:124. doi: 10.1186/s40824-023-00456-w (PMC10688116; doi:10.1186/s40824-023-00456-w)
Supplement: Supplementary file 1 — Supplementary Material 1 [file 40824_2023_456_MOESM1_ESM.docx]

**Supplementray information**

Post-insertion technique to introduce targeting moieties in milk exosomes for targeted drug delivery

*Hochung Jang^1,2^, Hyosuk Kim^1^, Eun Hye Kim^1,3^, Geonhee Han^1,4^, Yeongji Jang^1,3^, Yelee Kim^1,3^, Jong Won Lee^1,4^, Sang Chul Shin^5^, Eunice EunKyeong Kim^1^, Sun Hwa Kim^1,4*^ and Yoosoo Yang^1,2*^*

*Corresponding author:

Sun Hwa Kim, Ph.D. (S.H. Kim)

Tel: +82-2-958-6639; fax: +82-2-958-5909; e-mail: [sunkim@kist.re.kr](mailto:sunkim@kist.re.kr)

Yoosoo Yang, Ph.D. (Y. Yang)

Tel: +82-2-958-6655; fax: +82-2-958-5909; e-mail: [ysyang@kist.re.kr](mailto:ysyang@kist.re.kr)


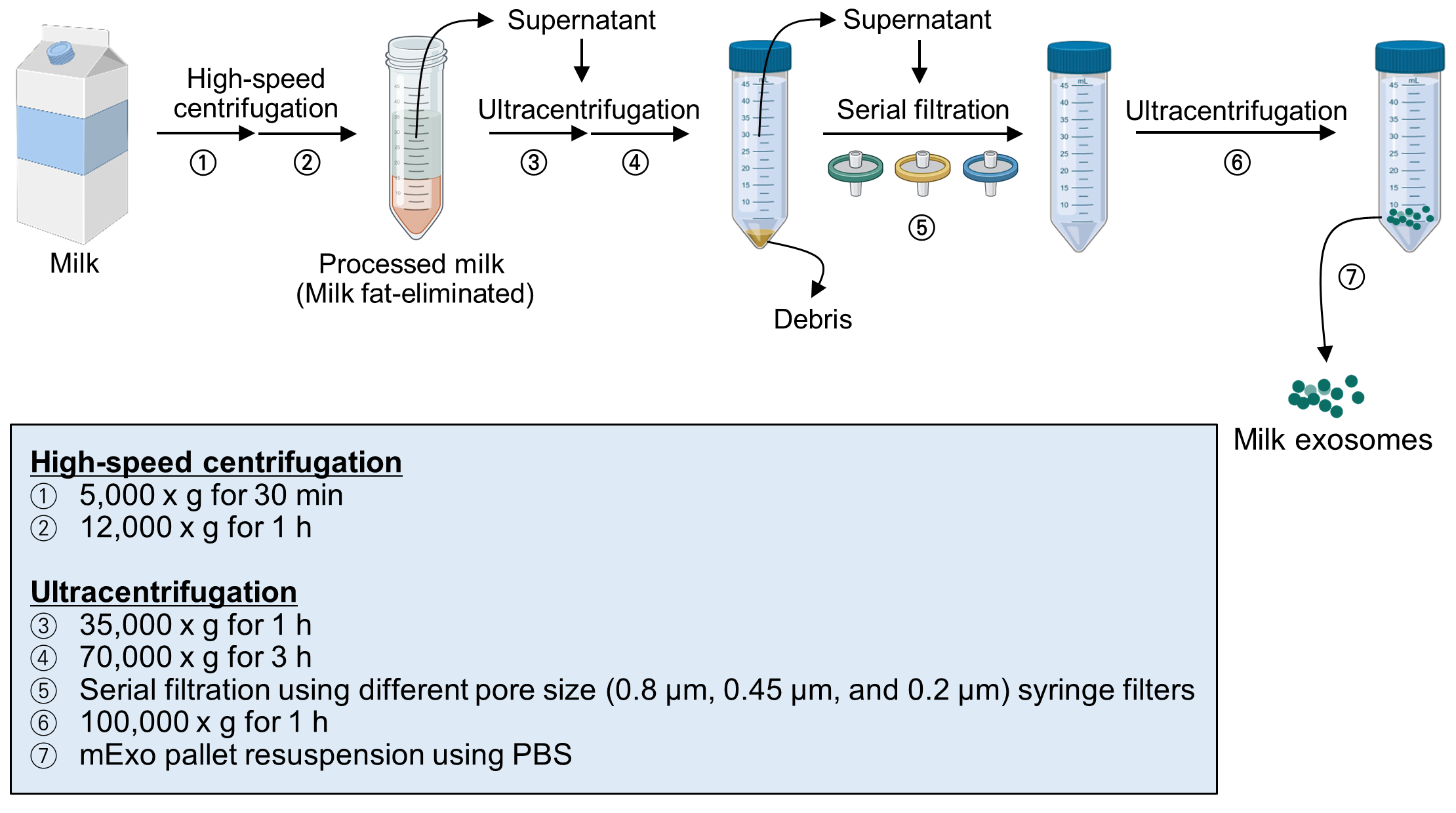


**Fig. S1. Schematic illustration of mExo isolation process.** The optimized mExo isolation protocol is composed of two steps; i) high-speed centrifugation step and ii) ultracentrifugation step with serial filtration.

**
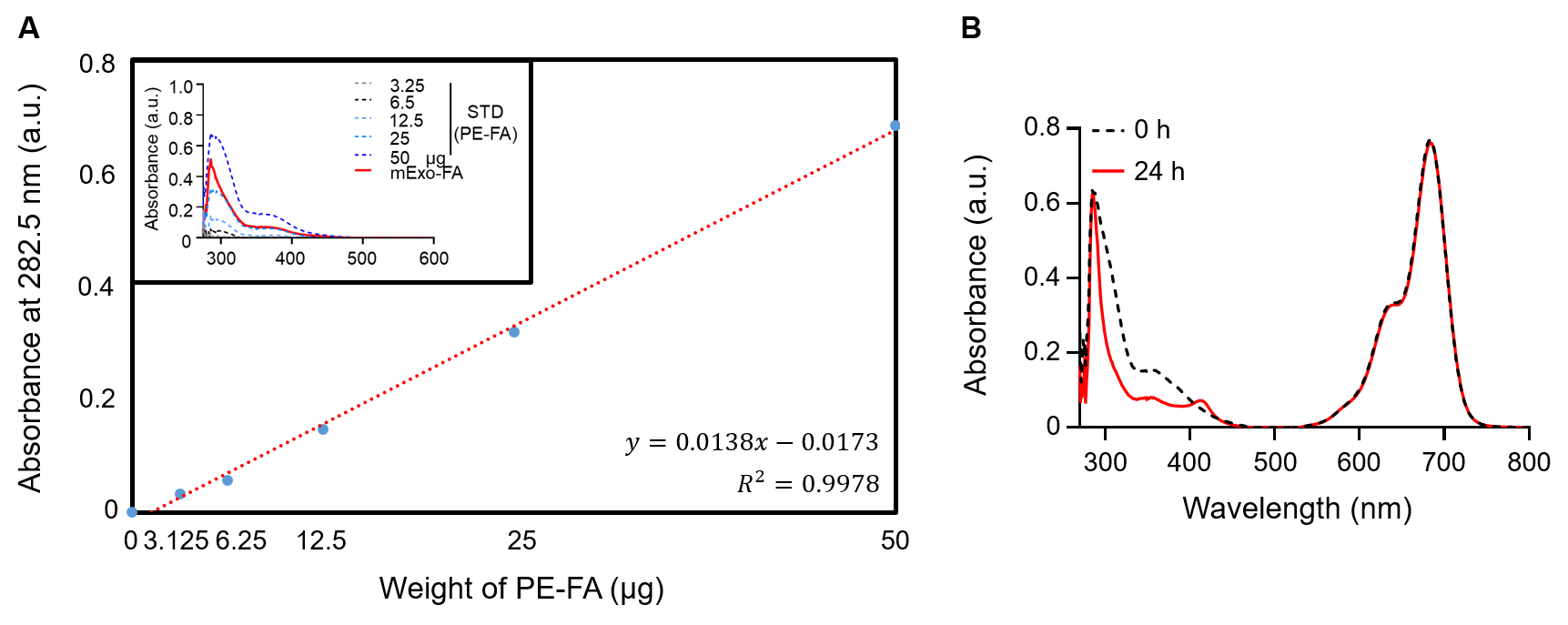
**

**Fig. S2. Post-insertion efficiency of FA and serum stability of F675-labeled mExo-FA.** (A) Standard calibration curve of FA (Peak at 282.5 nm) obtained from UV/Vis absorbance spectra. (B) Serum stability of F675-labeled mExo-FA. The absorbance peak at approximately 680 nm (F675 dye) and 282.5 nm (FA) indicates that FA insertion was stably maintained in mExo during serum incubation.


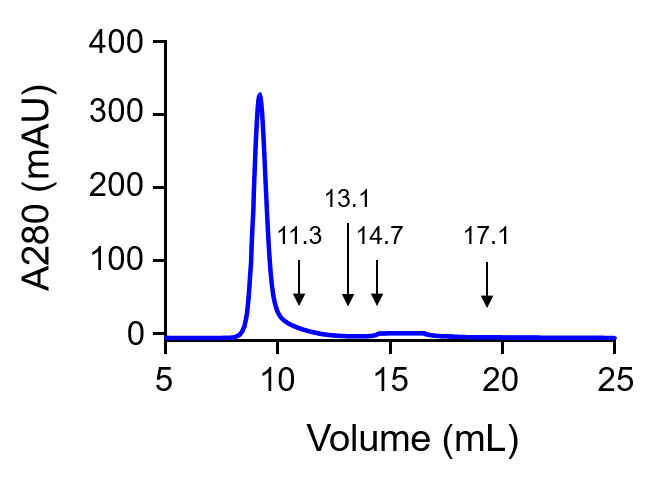


**Fig. S3. Quality check of exosome isolated from milk.** The gel filtration elution peak profile of exosome from size exclusion chromatography (Superdex-200S (10/300) column, GE Healthcare). The reference markers used are thyroglobulin (669 kDa = 11.3 mL), ferritin (445 kDa = 13.1 mL), b-amylase (200 kDa 14.7 mL), and albumin (66 kDa = 17.1 mL). The exosome and molecular weight markers (Sigma) were prepared separately in the buffers containing 1X PBS buffer.


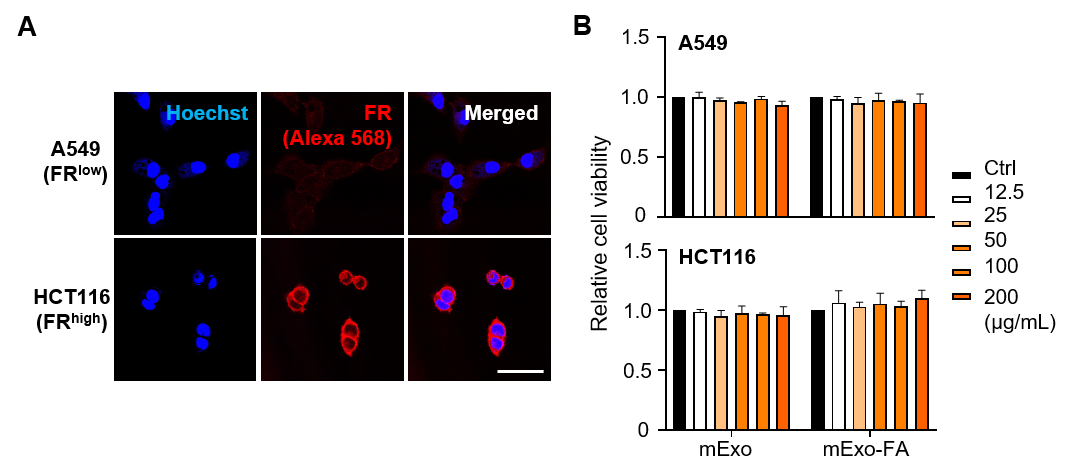


**Fig. S4. FR expression in A549 and HCT116 cell lines and *in vitro* cytotoxicity of mExo-FA.** (A) Confocal images for confirming FR expression in A549 and HCT116 cell lines. Alexa 568 (red) signals indicate the FR expression in each cell. Scale bar: 50 μm. (B) CCK8 assays to assess relative cell viability after 24 h incubation with various concentrations (12.5, 25, 50, 100, and 200 μg/mL) of mExo and mExo-FA (upper graph for A549, lower graph for HCT116).

**
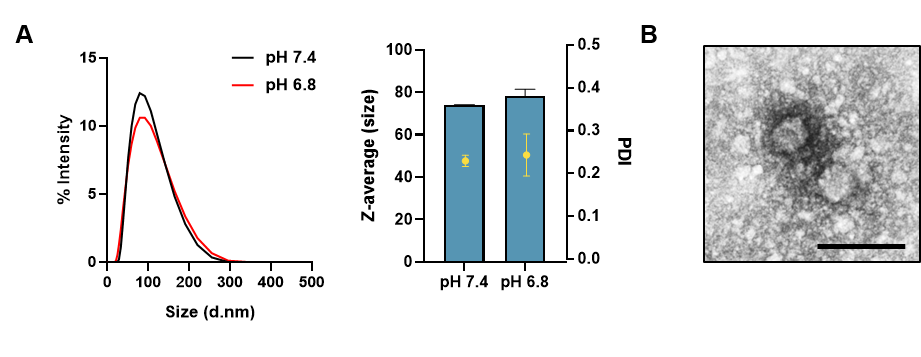
**

**Fig. S5. Particle size analysis of mExo in acidic (pH 6.8) and neutral (pH 7.4) conditions.**

**
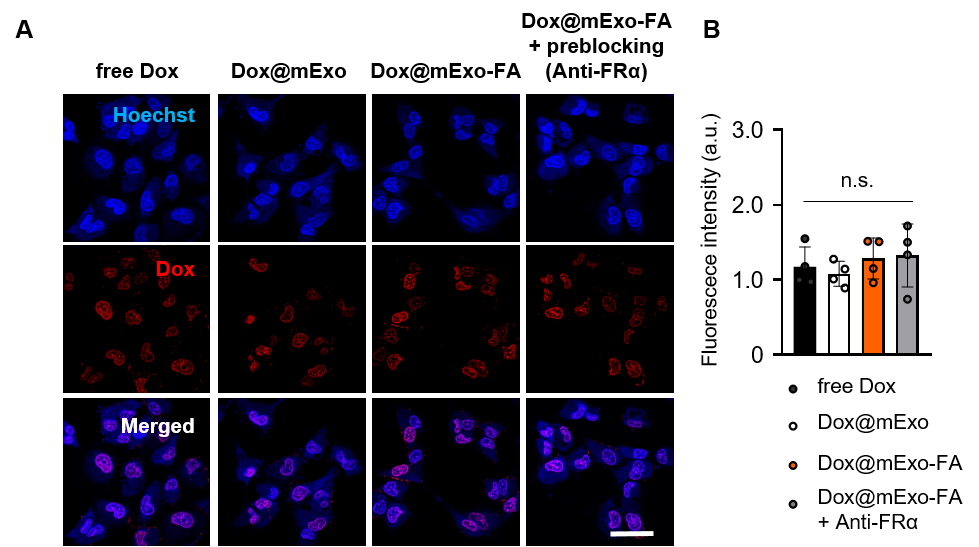
**

**Fig. S6.** **mExo-FA-mediated Dox delivery into A549 cells.** (A) Confocal images showing cellular uptake of Dox by Dox@mExo-FA in A549 cells (based on 1 μg/mL of Dox concentration). Scale bar: 50 μm. (B) Quantified graph indicating intracellular fluorescence intensity of Dox from (C).


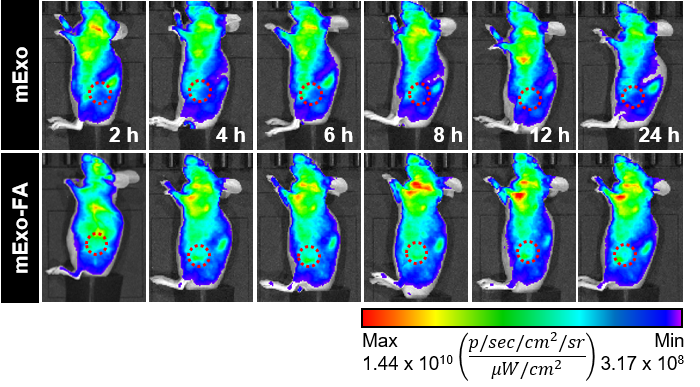


**Fig. S7. Whole-body fluorescence imaging after injection of mExo-FA in tumor-bearing mice.** Representative whole-body fluorescence images of HCT116 tumor-bearing mice at various time points (2, 4, 6, 8, 12, and 24 h after 200 μg of mExo/mExo-FA injection). The red dotted circles indicate the tumor site.
